# Rolipram, a PDE4 Inhibitor, Enhances the Inotropic Effect of Rat Heart by Activating SERCA2a

**DOI:** 10.3389/fphar.2019.00221

**Published:** 2019-03-22

**Authors:** Huili Huang, Ming Xie, Li Gao, Wenhui Zhang, Xiaojia Zhu, Yuwei Wang, Wei Li, Rongrong Wang, Kesu Chen, Mohamed Boutjdir, Long Chen

**Affiliations:** ^1^ National Standard Laboratory of Pharmacology for Chinese Materia Medica, School of Pharmacy, Nanjing University of Chinese Medicine, Nanjing, China; ^2^ Dalian Institute of Chemical Physics, Dalian, China; ^3^ Chinese Academy of Sciences Biomedical Innovation Institute of China Medical City, Taizhou, China; ^4^ Department of Respiratory, Inpatient Wards for Senior Cadres, Nanjing General Hospital of Nanjing Military Command Region, Nanjing, China; ^5^ VA New York Harbor Healthcare System, New York, NY, United States; ^6^ State University of New York Downstate Medical Center, New York, NY, United States; ^7^ NYU School of Medicine, New York, NY, United States; ^8^ Institute of Chinese Medicine of Taizhou China Medical City, Taizhou, China

**Keywords:** PDE4, rolipram, P-V loop, Ca^2+^ transient, SERCA2a

## Abstract

This study was designed to investigate the hemodynamic effect of rolipram, a phosphodiesterase type 4 (PDE4) inhibitor, in normal rat hearts both *in vivo* and *in vitro* and its underlying mechanism. The pressure-volume loop, isolated heart, and Ca^2+^ transients triggered by field stimulation or caffeine were used to analyze the hemodynamic mechanism of rolipram. The results demonstrated that rolipram (3 mg/kg, ip) significantly increased the *in vivo* rat heart contractility by enhancing stroke work, cardiac output, stroke volume, end-systolic volume, end-diastolic volume, end-systolic pressure, heart rate, ejection fraction, peak rate of rise of left pressure (+dp/dt_max_), the slopes of end-systolic pressure-volume relationship (slope of ESPVR) named as left ventricular end-systolic elastance, and reduced the slopes of end-diastolic pressure-volume relationship (slope of EDPVR). Meanwhile, the systolic blood pressure, diastolic blood pressure, and pulse pressure were significantly enhanced by rolipram. Also, rolipram deviated normal ventricular-arterial coupling without changing the arterial elastance. Furthermore, rolipram (0.1, 1, 10 μM) also exerted positive inotropic effect in isolated rat hearts by increasing the left ventricular development pressure, and +dp/dt_max_ in non-paced and paced modes. Rolipram (10 μM) increased the SERCA2a activity, Ca^2+^ content, and Ca^2+^ leak rate without changing diastolic Ca^2+^ level. Rolipram had significant positive inotropic effect with less effect on peripheral vascular elastance and its underlying mechanism was mediated by increasing SERCA2a activity. PDE4 inhibition by rolipram resulted in a positive inotropic effect and might serve as a target for developing agents for the treatment of heart failure in clinical settings.

## Introduction

Over 60 cyclic nucleotide phosphodiesterase (PDE) isozymes have been grouped into 11 classes based on their hydrolysis substrates and biochemical features (Knight and Yan, 2012). Of the 11 PDE families, at least 7 members, which are PDE1, 2, 3, 4, 5, 8, and 9, appear to be expressed in the myocardium (Kim and Kass, 2017). PDE1, 2, and 3 hydrolyze both cAMP and cGMP, PDE4 and 8 hydrolyze cAMP only, and PDE5 and 9 only hydrolyze cGMP (Knight and Yan, 2012). Except PDE9, all other six members involve the cardiac physiological and pathological activities (Miller and Yan, 2010). PDE3 inhibitors which further stimulate the force or frequency of the heart have been serving as inotropic agents for treating acute heart failure (Miller and Yan, 2010). With an increased risk of mortality for the chronic use of PDE3 inhibitors, the interest for developing the PDE inhibitors as the treatment of chronic heart failure has been reduced in the mid-1990s (Eschenhagen, 2013). However, due to the many more PDE subtypes and their inhibitors that have been discovered, the interests for developing potential drugs targeting PDEs have been reignited (Eschenhagen, 2013). Specifically, the selective PDE5 inhibitors have been used for the treatment of erectile dysfunction and pulmonary hypertension, and a selective PDE4 inhibitor (roflumilast) was recently licensed for the treatment of chronic obstructive lung disease (Maurice et al., 2014). All these diseases treated by the selective PDE5 or PDE4 inhibitors are associated with increased cardiovascular risk (Eschenhagen, 2013). Meanwhile, the clinical and basic studies for treating heart failure based on targets of selective PDEs are still ongoing.

The small, highly diffusible molecule, cAMP exerts multiple, discrete receptor-specific responses in the same cells (Fertig and Baillie, 2018). Such multiple responses of cAMP are based on compartmentalization of cAMP signaling by restricting the number and identity of PKA-phosphorylated substrates (Zaccolo and Pozzan, 2002) and the fine control by the subcellular localization of PDEs that degrade cAMP (Fertig and Baillie, 2018). Among the six PDE families which are expressed in the myocardium, PDE4 appears to be predominantly in the heart (Eschenhagen, 2013). More than 20 isoforms belong to four different PDE4 genes, PDE4A, 4B, 4C, and 4D, with PDE4A, 4B, and 4D detected in the hearts (Kostic et al., 1997), with PDE4 and D5 interacting with β-arrestin (Bolger et al., 2003), PDE4B and 4D with the cardiac L-type Ca^2+^ channel (Leroy et al., 2011), PDE4B with ryanodine receptor (RyR) (Fertig and Baillie, 2018), PDE4D with SERCA2a (Beca et al., 2011), PDE4D3 with slow delayed rectifier potassium current I_KS_ channel (Terrenoire et al., 2009; Parks et al., 2014). The cellular integrated function of PDE4 is based on the distribution of the localized PDE4 subtypes and cAMP levels which are affected by adrenergic stimulation (Fertig and Baillie, 2018). Therefore, the functions of PDE4 in the myocardium may differ by sex (Parks et al., 2014), age (Park et al., 2012), species (Richter et al., 2011), cell types (Molina et al., 2014), and pathological status (Zheng et al., 2014).

The studies on cellular, biochemical, molecular, and structural changes in the heart tissue induced by PDE4 inhibitors have been extensively performed (Kim and Kass, 2017). The studies of non-selective PDE4 inhibitor on cardiac hemodynamics both *in vivo* and *in vitro* are still lacking. This study aimed at investigating the effects of rolipram, a PDE4 inhibitor, on the hemodynamics from both *in vivo* and isolated heart and the underlying cellular Ca^2+^ handling mechanism. In addition, the present study addresses the potential clinical benefit of PDE4 inhibitor for the treatment of heart failure and provides additional information for understanding the precise regulation of subtypes of PDE4 in the heart.

## Materials and Methods

### Chemicals

Rolipram with a purity of more than 99.56% was purchased from MedChemExpress (MCE) in Shanghai (China) with the catalog number of HY-16900. All other chemicals were purchased from Sigma-Aldrich (USA). Rolipram was dissolved in DMSO at room temperature and used on the same day.

### *In vivo* Left Ventricular and Arterial Hemodynamic Parameters Recording

The investigation conformed to the Guide for the Care and Use of Laboratory Animals published by the US National Institutes of Health (NIH publication No. 85-23, revised 1996). Male Sprague Dawley rats (300–350 g) (from the laboratory animal center of Nanjing University of Chinese Medicine) were used in this study. Experiments were performed as previously described (Li et al., 2017). Briefly, the rats were anesthetized by 20% urethane (5 ml/kg, ip) and their right carotid arteries were inserted with two pressure and volume microsensors’ catheter (MILLAR, SPR-901, 840-8,188, Houston, USA) connected to a PowerLab 4/30 data acquisition system (AD Instruments, PowerLab4/30, Australia). The two pressure sensors at the microtip of catheter were placed in the aorta or left ventricle for each and used to measure the pressures of arterial or left ventricular pressures simultaneously. Rolipram (3 mg/kg) dissolved in DMSO less than 0.5 ml was injected intraperitoneally. DMSO (0.5 ml) had no measurable effects on the hemodynamic parameters studied. The control values were recorded after the hemodynamic parameters remained constant and before injection of rolipram. Rolipram’s effects were recorded after all parameters reached constant values with usually longer than 30 min. The pressure and volume sensors in the left ventricle were set to analyze the pressure-volume relationship (P-V loop). The hemodynamic parameters were analyzed with Labchart 8 from AD Instruments including stroke work, cardiac output, heart rate, stroke volume, end-systolic volume, end-diastolic volume, end-systolic pressure, end-diastolic pressure, ejection fraction, and +dp/dt_max_ (peak rate of rise of left ventricular pressure), Slope of linear regression’ ESPVR (end-systolic pressure-volume relationship) which is also named as left ventricular end-systolic elastance, Slope of linear regression’ EDPVR (end-diastolic pressure-volume relationship). Furthermore, the systolic blood pressure, diastolic blood pressure, pulse pressure, arterial elastance which is the ratio of end-systolic pressure to stroke volume, and ventricular-arterial coupling were analyzed.

### *In vitro* Intraventricular Pressure Recording From Isolated Rat Hearts

The selection and anesthesia of rats for *ex vivo* experiments were the same as that of *in vivo* experiment described above and the methods used were described previously (Li et al., 2017). Briefly, the hearts were then perfused in the Langendorff non-recirculating mode, at a perfusion pressure of 80 mmHg, with a perfusion solution (37°C) containing (in mM): NaCl 117, KCl 5.7, CaCl_2_ 1.8, MgCl_2_ 1.7, NaHCO_3_ 4.4, NaH_2_PO_4_ 1.5, HEPES 20, Glucose 11 gassed with 95% O_2_ plus 5% CO_2_ (pH 7.4 with NaOH). Rolipram was dissolved in DMSO and then transferred to perfusion solutions to reach the final concentrations of 0.1, 1, and 10 μM, respectively. The control perfusion solutions contained the same concentrations of DMSO as that in rolipram groups. During the experiments, these different concentrations of rolipram from low to high concentration order were added to the perfusion solution and infused *via* retrograde perfusion of the coronary artery. The hemodynamic parameters of control or rolipram (0.1, 1, 10 μM) were recorded when isolated heart contraction reached constant level with rolipram perfusions longer than 10 min for each concentration. Their intraventricular pressures under non-isovolumic condition of contraction were measured by a pressure and volume microtip catheter (MILLAR, SPR-901, 840-8,188, Houston, USA) inserted into the left ventricle *via* the left atrium. The hearts were beating spontaneously (non-paced) or stimulated at a rate of 4 Hz (paced). The paced isolated hearts were stimulated between left and right atrium at 5 V. Left ventricular developed pressure was calculated as the difference between the peak systolic pressure and end-diastolic pressure. Also, the heart rate only in non-paced setting and + dp/dt_max_ were calculated.

### Ca^2+^ Transient Recording of Adult Rat Left Ventricular Myocytes Triggered by Field Stimulation

For isolation of adult rat left ventricular myocytes, the rat ventricular myocytes were obtained by enzymatic dissociation as previously described (Li et al., 2017). The excised whole heart was first perfused at 37°C with a perfusion solution (37°C) containing (in mM): NaCl 117, KCl 5.7, CaCl_2_ 1.8, MgCl_2_ 1.7, NaHCO_3_ 4.4, NaH_2_PO_4_ 1.5, HEPES 20, Glucose 11 gassed with 95% O_2_ plus 5% CO_2_ (pH 7.4 with NaOH). The heart was then perfused with the same buffer with the addition of 2.0 mg/ml collagenase type II and 0.1 mg/ml Protease Type for 50 min. Following removal of both atria and the right ventricle, the left ventricular myocytes were gently separated with forceps in the buffer without collagenase. Rod-shaped non-contracting cells with clear striations were used.

For Ca^2+^ transient recording triggered by field stimulation, adult rat left ventricular myocytes were loaded with the membrane-permeable acetoxymethyl ester form of the fluorescent Ca^2+^ indicator Fluo-4 AM (5 μM) for 30 min at 37°C. Fluo-4 was excited at 488 nm (Lambda DG-4, Sutter instruments, USA) and emitted fluorescence measured with a 515-nm-long pass filter. The region of interest was restricted to a single cell with the aid of an adjustable window. The Ca^2+^ transient amplitudes were calculated as a difference of the peak and diastolic fluorescent values. Background fluorescence levels were used to correct raw fluorescence data. Ca^2+^ spark fluorescent intensity of Fluo-4 loaded in myocytes was recorded at imaging frequency of 100 Hz during electrical pacing (1 Hz, 15 V, alternating polarities) with two platinum electrodes. The extracellular solution contained (in mM): NaCl 117, KCl 5.7, NaHCO_3_ 4.4, MgCl_2_ 1.7, HEPES 20, Glucose 20, Taurine 20, CaCl_2_ 1.8, pH 7.4 with NaOH. After the constant imaging of control was recorded, rolipram (10 μM) in the extracellular solution was perfused for longer than 5 min and then its constant Ca^2+^ transient effect was recorded. Images were recorded with an ANDOR ZYLA-5.5-CL3 CCD camera (AndorTechnologe, EU) connected to an inverted microscope (Olympus IX53, Olympus, Tokyo, Japan) that was synchronized by a real-time analog-digital processor unit and Meta fluor acquisition software (64-bit, version 7.8.10.0, Molecular Device, USA).

The decaying phase of Ca^2+^ transient involves two components, which are a fast component dominated by sarcoplasmic reticulum (SR) Ca^2+^-ATPase type 2a (SERCA2a) and slow component dominated mainly by Na^+^/Ca^2+^ exchanger (NCX) and sarcolemmal Ca^2+^-ATPase (PMCA). Isolation of two components was analyzed by the theory and method of plasma concentration-time curve with two exponential fitting following intravenous administration of a drug in human.

### Functional Isolations of Na^+^/Ca^2+^ Exchanger (NCX) and Sarcolemmal Ca^2+^-ATPase (PMCA) From the Decaying Phase of Caffeine-Evoked Ca^2+^ Transients

The decaying phase of Ca^2+^ transient by field stimulation is mediated mainly by SERCA2a, NCX, and PMCA. The NCX and PMCA control the decaying phase of caffeine-evoked Ca^2+^ transient in normal Tyrode’s solution (NT) in which caffeine prevents the effect of SERCA2a on SR Ca^2+^ concentration gradient formation by widely opening RyR2. Also, PMCA function can be isolated from decaying phase of the caffeine-evoked Ca^2+^ transient in the condition of Na^+^, Ca^2+^-free solution which deactivates NCX activity. The rate constants of decline of caffeine-evoked Ca^2+^ transient in normal Tyrode’s solution and Na^+^, Ca^2+^-free solution were used to analyze the functions of mixed NCX plus PMCA and PMCA alone. The detailed methods were previously described (Choi and Eisner, 1999; Maczewski and Mackiewicz, 2008; Schulte et al., 2016). Briefly, immediately after stopping the field stimulation when the steady Ca^2+^ transient was obtained, caffeine (20 mM) was perfused to myocytes in normal Tyrode’s solution to obtain the decaying rate constant of combined activity of NCX and PMCA. To calculate the decaying rate constant of PMCA alone, Ca^2+^ leak rate, and Ca^2+^ content, Na^+^,Ca^2+^-free solution was perfused before caffeine (20 mM) perfusion. The normal Tyrode’s solution contained (in mM): NaCl 134, KCl 4, MgCl_2_ 1, HEPES 10, Glucose 11, CaCl_2_ 1, adjusted to pH 7·4 with NaOH. The Na^+^, Ca^2+^-free solution contained (in mM): LiCl 130, HEPES 10, Glucose 11, MgCl_2_ 1, KCl 4, adjusted to pH 7.4 with KOH. For control experiment, normal Tyrode’s solution was used to record functional isolations of NCX and PMCA. Rolipram (10 μM) was added to recording chamber for at least 10 minutes to maximize its effects on NCX and PMCA. The effects of control or rolipram on combined function of NCX plus PMCA or PMCA only were performed in different group cells and *t*-test was used to analyze significant differences.

### Statistical Analyses

All values are expressed as mean ± SEM. *t*-test and one-way ANOVA were used as appropriate and data analysis was performed using SPSS 11.0. Differences with *p* < 0.05 were deemed statistically significant.

## Results

### Rolipram Increased Left Ventricular Inotropy of Rat Heart *in vivo*

**Figure 1:**
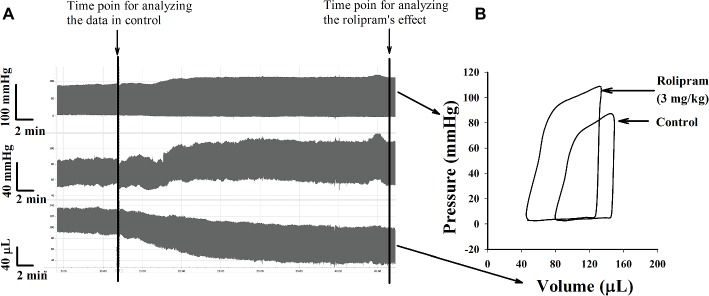
**(A)** Original simultaneous recordings of left ventricular pressure, aortic blood pressure and left ventricular volume versus time before and after rolipram (3 mg/kg, ip) to an anesthetized rat. **(B)** The derivative steady-state P-V loops of left ventricular pressure versus its volume before and after rolipram (3 mg/kg, ip) at marked time points.

**Figure 2:**
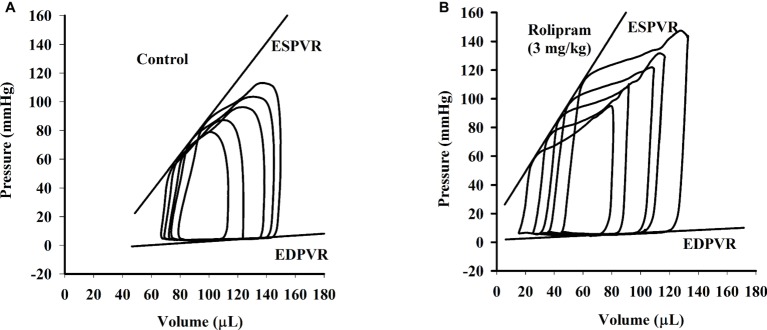
The continuous multi P-V loops’ recordings when the rat inferior vena cava was blocked before **(A)** and after **(B)** intravenous injection of rolipram (3 mg/kg) to an anesthetized rat. The slope of ESPVR is formatted by linear regression of multi end-systolic pressures and the slope of EDPVR by linear regression of multi end-diastolic pressures.

**Figure 3:**
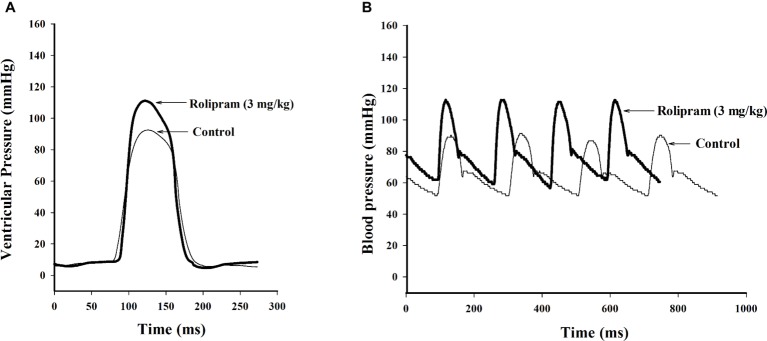
Original simultaneous recordings of left ventricular pressures **(A)** and aortic blood pressures **(B)** versus time course before and after rolipram (3 mg/kg, ip) to an anesthetized rat.

To examine the hemodynamic effects of rolipram on the rat heart and peripheral vessels, the pressure-volume loop (P-V loop) technique was used to measure the left ventricular pressure-volume and aortic pressure simultaneously. Rolipram (3 mg/kg, ip) significantly enhanced the stroke work, cardiac output, stroke volume, end-systolic pressure, heart rate, ejection fraction, and +dp/dt_max_, and decreased the end-systolic volumes, end-diastolic volume compared with control (*p* < 0.05, *n* = 6) without significantly changing end-diastolic pressure. Also, the slope of ESPVR (Ees) was increased and the slope of EDPVR was reduced as shown in Table 1. Figure 1 shows the representative original simultaneous recordings of the left ventricular pressure, volume, and aortic pressure and the derivative relationship of P-V loop. The higher and wider P-V loop induced by rolipram indicates increased left ventricular pressure and stroke volume. Moreover, the steeper slope of ESPVR (Ees) induced by rolipram indicates the increased contractile function which is independent of preload. The smoother slope of EDPVR reflects decreased diastolic stiffness compared with control as shown in Figure 2. The arterial hemodynamic effective changes indicated that rolipram increased the systolic blood pressure, diastolic blood pressure, and pulse pressure as shown in Table 2. Moreover, rolipram decreased the ventricular-arterial coupling, indicating rolipram had inotropic effect without significant arterial elastance change. Figure 3 shows the simultaneous recordings of rat left ventricular and aortic pressure versus time course.

**Table 1:** Pressure-volume relationship analysis in normal and rolipram (3 mg/kg) treated anesthetized rats.

Kinetic parameters	Control	Rolipram
Stroke work (mmHg μl)	5,970 ± 168.2	9,664 ± 214.4^*^
Cardiac output (μl/min)	20,260 ± 538.6	32,442 ± 819.2^*^
Stroke volume (μl)	68 ± 0.7	88 ± 1.0^*^
end-systolic volume (μl)	79 ± 1.3	46 ± 2.7^*^
end-diastolic volume (μl)	147 ± 1.7	133 ± 3.7^*^
end-systolic pressure (mmHg)	88 ± 2.0	110 ± 2.5^*^
end-diastolic pressure (mmHg)	2.8 ± 0.1	2.5 ± 0.3
Heart rate (bpm)	298 ± 6.9	370 ± 5.8^*^
Ejection fraction (%)	46 ± 0.4	66 ± 1.1^*^
+dP/dt_max_ (mmHg/s)	4,306 ± 327.8	9,005 ± 756.7^*^
Slope of ESPVR (Ees)	1.246 ± 0.030	1.699 ± 0.031^*^
Slope of EDPVR	0.108 ± 0.010	0.080 ± 0.008^*^

+dp/dt_max_, Peak rate of rise of left pressure; Slope of ESPVR, the slopes of end-systolic pressure-volume relationship or left ventricular end-systolic elastance (Ees); Slope of EDPVR, the slopes of end-diastolic pressure-volume relationship; mean ± SEM, *n* = 6.

* p < 0.05 vs respective control. All parameters for rolipram were under condition of intraperitoneal injection except Ees and slope of EDPVR for rolipram by intravenous injection.

**Table 2:** Effects of rolipram (3 mg/kg, ip)on aortic blood pressure and ventricular-arterial coupling in anesthetized rats.

Kinetic parameters	Control	Rolipram
Systolic blood pressure (mmHg)	90 ± 2.4	116 ± 1.8^*^
Diastolic blood pressure (mmHg)	48 ± 1.4	61 ± 3.3^*^
Pulse pressure (mmHg)	42 ± 2.6	55 ± 2.3^*^
Ea (mmHg/μl)	1.292 ± 0.027	1.264 ± 0.036
Ea/Ees	1.041 ± 0.037	0.746 ± 0.032^*^

Ea, arterial elastance; Ea/Ees: Ventricular-arterial coupling; mean ± SEM, *n* = 6.

* p < 0.05 vs respective control.

### Rolipram Increased Left Ventricular Inotropy of Isolated Rat Hearts

**Figure 4:**
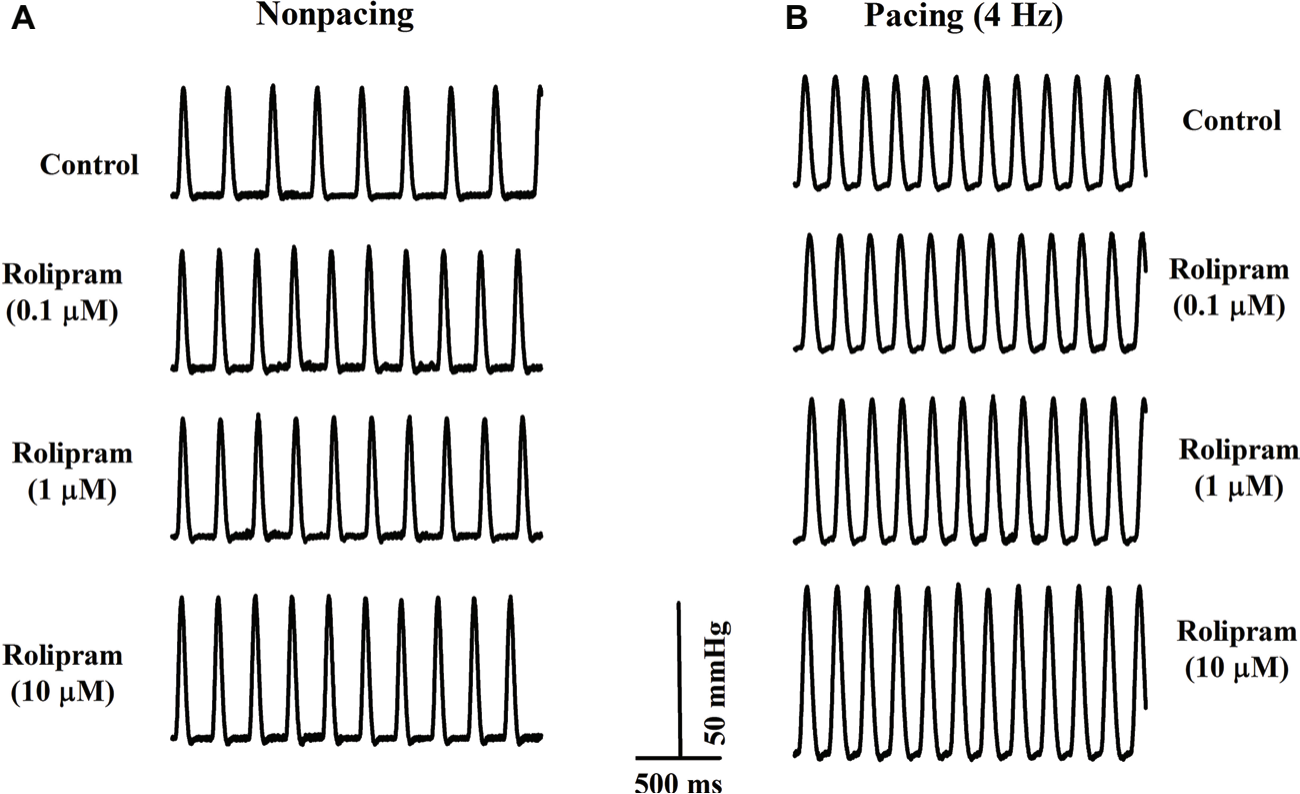
Representative rat left ventricular pressure curves from the isolated rats in non-paced **(A)** and paced modes **(B)** before and after perfusion of rolipram (0.1, 1, 10 μM) with the same concentrations of DMSO.

To further investigate the direct effect of rolipram on the heart, the isolated non-paced and paced rat hearts were used to analyze the inotropic and chronotropic effects of rolipram. The pacing was used to rule out the frequency-induced inotropic effect and 4-Hz pacing was chosen to match the non-paced heart rate as shown in Figure 4. As documented in Table 3, rolipram (0.1, 1, 10 μM) increased the left ventricular developed pressure, heart rate, and +dp/dt_max_ in non-paced mode in a concentration-dependent manner. In paced mode, rolipram (0.1, 1, 10 μM) also increased in a concentration-dependent manner the left ventricular developed pressure and +dp/dt_max_ with a fixed heart rate of 240 beats per min.

**Table 3:** Effects of rolipram in isolated rat hearts in the conditions of nonpacing and pacing modes.

	Nonpacing			Pacing (4 Hz)		
	LVDP (mmHg)	HR (bpm)	+dp/dt_max_ (mmHg/s)	LVDP (mmHg)	HR (bpm)	+dp/dt_max_ (mmHg/s)
Control	39 ± 2.3	177 ± 3.6	991 ± 21.0	38 ± 2.4	240	1,230 ± 24.7
Rolipram (0.1 μM)	42 ± 2.1^*^	189 ± 4.1^*^	1,027 ± 35.8	44 ± 2.6^*^	240	1,308 ± 34.4^*^
Rolipram (1 μM)	42 ± 2.3^*^	192 ± 3.8^*^	1,061 ± 34.9^*^	48 ± 2.4^*#^	240	1,465 ± 26.4^*#^
Rolipram (10 μM)	47 ± 2.9^*#△^	200 ± 4.1^*#△^	1,132 ± 35.8^*#△^	52 ± 2.2^*#^	240	1,655 ± 28.5^*#△^

LVDP: left ventricular developed pressure; HR: heart rate; +dP/dt_max_: peak rate of rise of left ventricular pressure. Rolipram was dissolved in DMSO before reaching the desired concentrations and control perfusion solution also contained the same concentration of DMSO as that in rolipram groups. Data are expressed as Mean ± SEM, *n* = 6. ^*^p < 0.05 vs control; ^#^p < 0.05 vs 0.1 μM groups; △*p* < 0.05 vs 1 μM groups in non-paced and paced modes respectively.

### Rolipram Enhanced the Ca^2+^ Uptake of Field Stimulation-Induced Ca^2+^ Transient by Facilitating SERCA2a Activity

**Figure 5:**
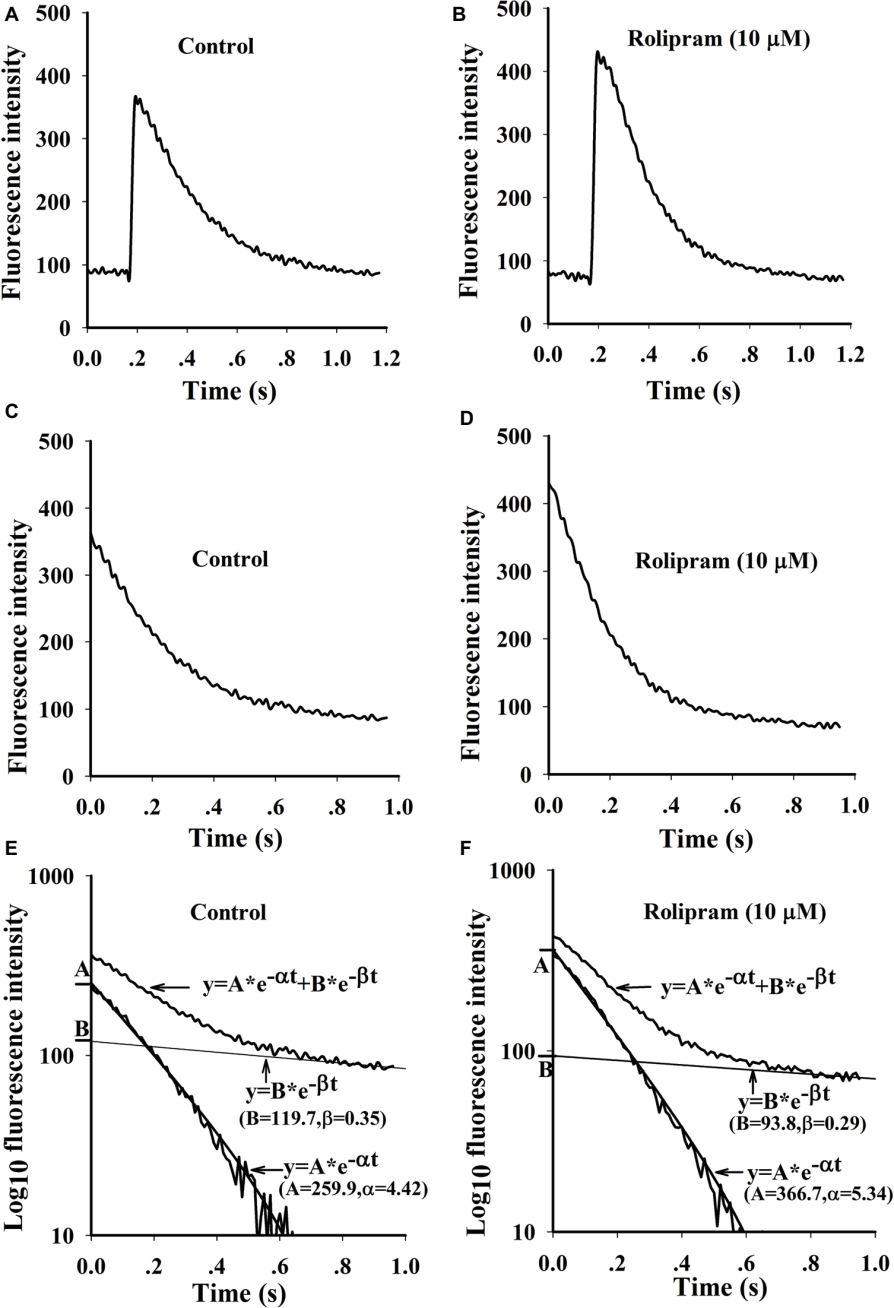
Representative curves of Ca^2+^ transients induced by field stimulation and the fittings by two exponential equations. **(A** and **B)** are original Ca^2+^ transient curves triggered by field stimulation before and after rolipram (10 μM). **(C** and **D)** are decaying phase curves of Ca^2+^ transients by deleting the fast rise phase before and after rolipram (10 μM). The decay phase curves and fittings are presented as the relationships of timing of ms in X axis to the log_10_ fluorescence intensities in Y axis before **(E)** and after rolipram (10 μM) **(F)**. The decaying phases are expressed as an equation of *y* = A*e^−αt^ + B*e^−βt^. The equation in fast component is described as *y* = A*e^−αt^ and slow one as *y* = B*e^−βt^.

Rolipram (10 μM) significantly enhanced the amplitude of Ca^2+^ transient from rat left ventricular myocytes without changing the diastolic baseline fluorescence intensity (proportional to cytoplasmic Ca^2+^ level) as shown in Table 4. The separated decaying phases were fitted by two exponential equations according to method of plasma concentration-time curve fitting following intravenous administration of a drug in human. The representative fitting curves with fast and slow components are presented in Figure 5. Rolipram (10 μM) remarkably enhanced the fast component rate constant (α) and decreased the slow component rate constant (β) as shown in Table 4, indicating that rolipram enhanced SERCA2a activity and reduced the combined activity of NCX and PMCA.

**Table 4:** Kinetic analysis of rolipram’s effecton Ca^2+^ transient induced by field stimulation in left ventricular myocytes.

Kinetic parameters	Control	Rolipram (10 μM)
I_baseline_ (%)	100 ± 3.5	102 ± 1.3
∆I_amplitude_ (%)	100 ± 6.0	112 ± 2.9^*^
α	5.20 ± 0.32	6.35 ± 0.83^*^
β	0.30 ± 0.03	0.26 ± 0.03^*^

I_baseline_(%): Normalized baseline fluorescence intensity of Ca^2+^ transient as 100% in control; ∆I_amplitude_ (%); Normalized fluorescence intensity amplitude of Ca^2+^ transient as 100% in control; α: rate constants of fast component mediated by SERCA2a activityin decaying phase of Ca^2+^ transient; β: rate constants of slow component mediated by NCX and PMCA activities in decaying phase of Ca^2+^ transient. Data are expressed as Mean ± SEM, Paired *t*-test was used, ^*^p < 0.05 compared with their respective controls, *n* = 6.

### Rolipram Had No Direct Effects on Combined NCX and PMCA Activities in Caffeine-Induced Ca^2+^ Transient

**Figure 6:**
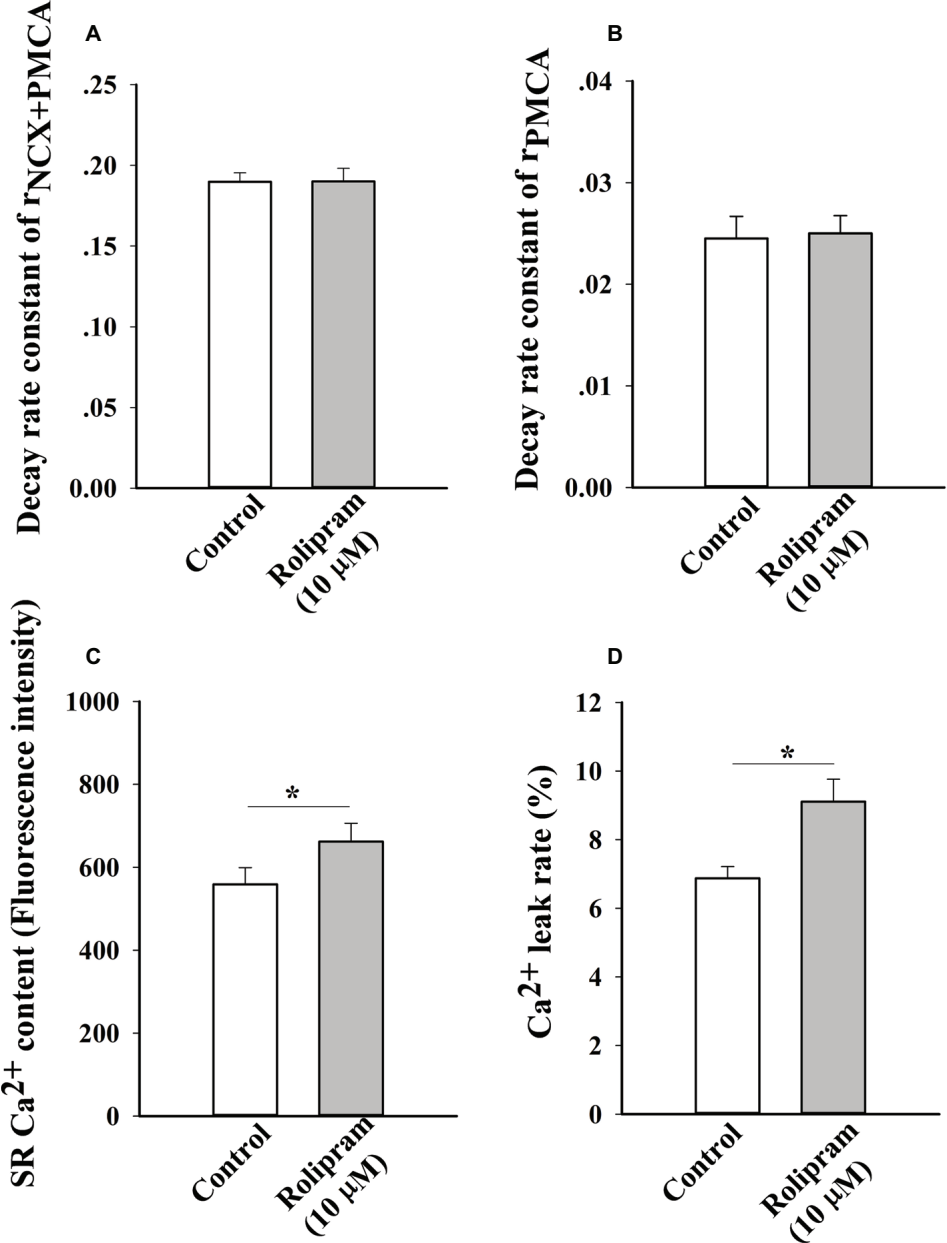
The histograms show the statistical results of decaying rate constants of Ca^2+^ extrusion by combined activity of NCX and PMCA **(A)**, PMCA alone **(B)**, Ca^2+^ contents **(C)** and Ca^2+^ leak rates of real leak value to Ca^2+^ content **(D)**. The *t*-test was used to analyze the data in control and rolipram-treated rat left ventricular myocytes. ^*^ *p* < 0.05 vs respective control, *n* = 11 for all groups.

**Figure 7:**
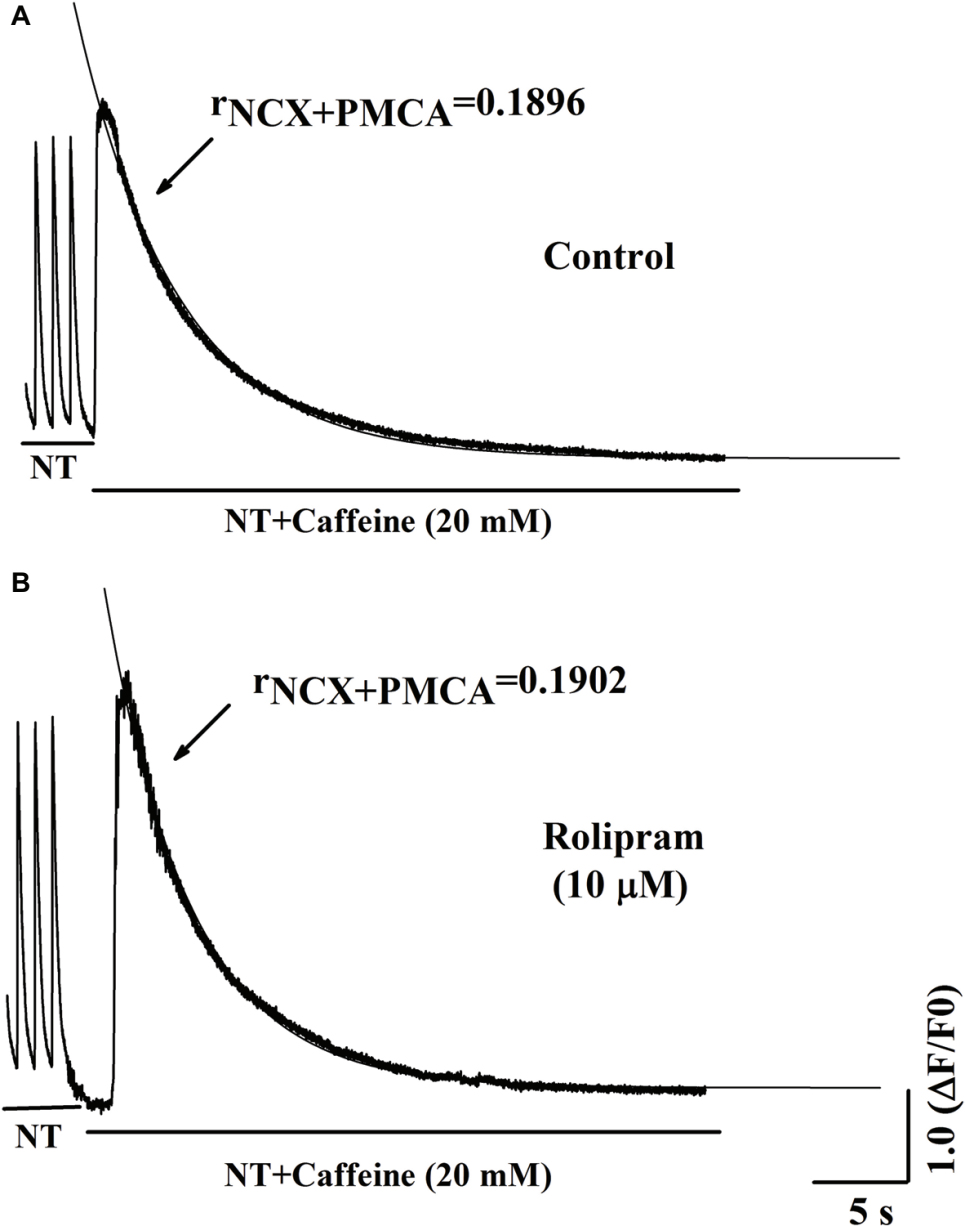
Representative curves of Ca^2+^ transient induced by caffeine in normal Tyrode’s solution (NT) before **(A)** and after **(B)** rolipram (10 μM). The r_NCX + PMCA_ is presented as decaying phase rate constant which is dominated by combined activity of NCX and PMCA to extrude Ca^2+^ to outside cell.

To further analyze the decreased rate constant of NCX and PMCA activities in field stimulation-induced Ca^2+^ transient, caffeine-induced Ca^2+^ transient experiment was performed. In this condition, SERCA2a activity was abolished due to the opening of RyR2 by caffeine and the decaying phase mainly was mediated by NCX and PMCA. The result in Figure 6A shows that rolipram (10 μM) did not significantly change the rate constant of combined activity of NCX and PMCA (r_NCX + PMCA_) from 0.190 ± 0.006 in control to 0.190 ± 0.008 (*p* < 0.05, *n* = 11). The representative curves of caffeine-induced Ca^2+^ transients in normal Tyrode’s solution before and after rolipram (10 μM) injection are shown in Figure 7.

### Rolipram Increased the Diastolic Ca^2+^ Leak Rate and SR Ca^2+^ Content Without Direct Effect on PMCA Activity in Caffeine-Induced Ca^2+^ Transient

**Figure 8:**
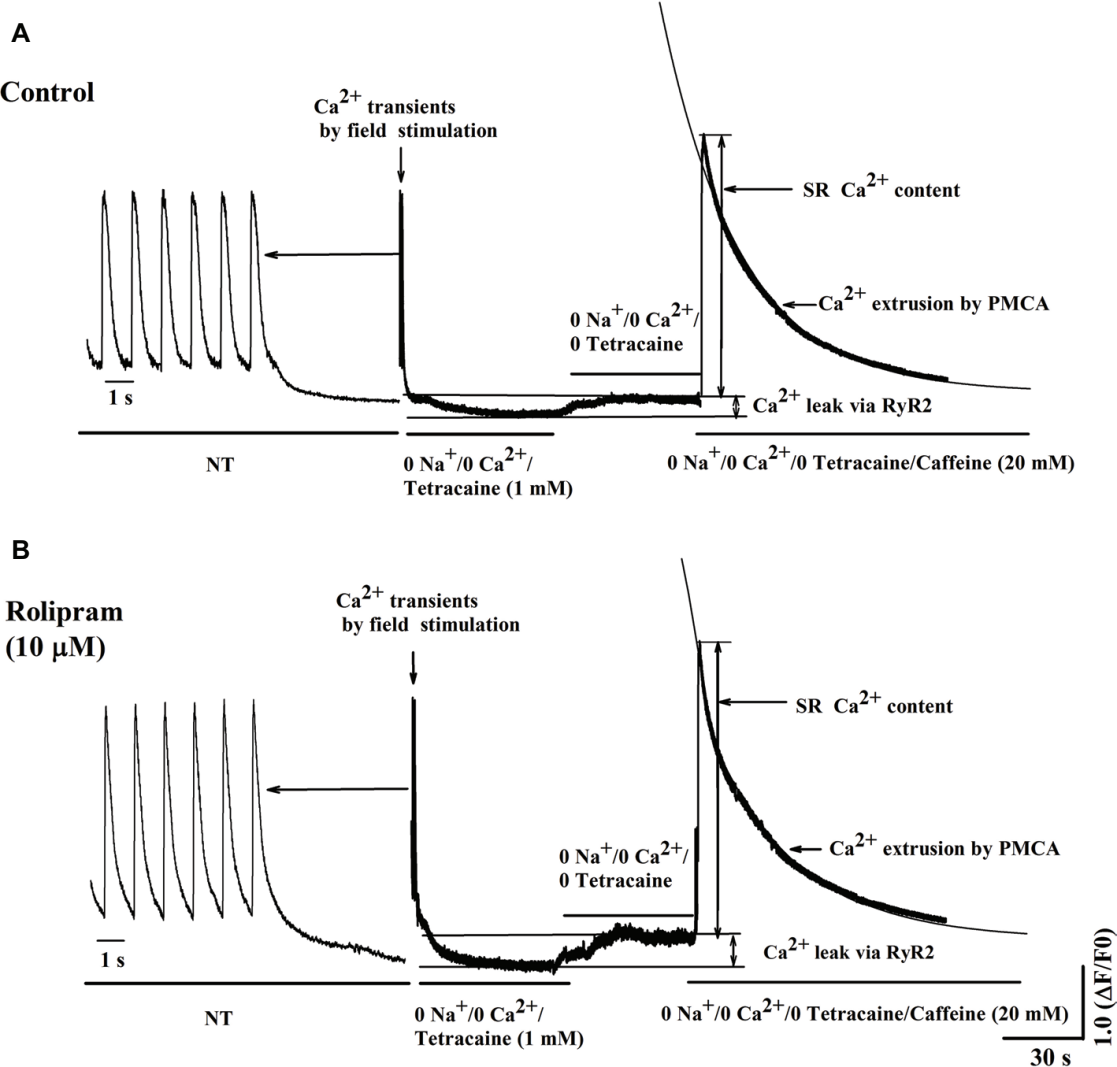
Representative curves of Ca^2+^ leak, SR Ca^2+^ content, and Ca^2+^ transient induced by caffeine before **(A)** and after **(B)** rolipram (10 μM) in the conditions of Na^+^ and Ca^2+^-free extracellular solution following steady field stimulation-induced Ca^2+^ transient in normal Tyrode’s solution (NT). The r_PMCA_ is presented as decaying phase rate constant which is dominated by activity of PMCA alone to extrude Ca^2+^ to outside cell.

To further isolate the activity of PMCA by rolipram from combined activity of NCX and PMCA, the Na^+^ and Ca^2+^-free extracellular solution was used to block the activity of NCX. In this condition, the Ca^2+^ transient decaying phase is mainly mediated by PMCA. Rolipram (10 μM) had no effect on PMCA activity based on insignificant rate constant (r_PMCA_) change from 0.025 ± 0.002 in control to 0.025 ± 0.002 (*p* < 0.05, *n* = 11) as shown in Figure 6B. However, rolipram (10 μM) significantly increased SR Ca^2+^ content from 559.0 ± 40.2 in control to 662.0 ± 44.2 (Figure 6C) (*p* < 0.05, *n* = 11) and enhanced the diastolic Ca^2+^ leak rate from 6.9% ± 0.3 in control to 9.1% ± 0.7 (Figure 6D) (*p* < 0.05, *n* = 11). The representative Ca^2+^ transient curves induced by caffeine in Na^+^ and Ca^2+^-free extracellular solution are presented in Figure 8.

## Discussion

Three subtypes of PDE4 (PDE4A, B, D) are expressed in rat heart (Fertig and Baillie, 2018). Although their fundamental mechanism involving hydrolysis of cAMP is clear, their functions, especially *in vivo*, are not dissected due to their differential compartment distribution and basal level of hydrolysis substrates (Miller and Yan, 2010). In this P-V loop study, we underlined that rolipram, a PDE4 inhibitor, significantly increased the rat heart rate and contractility based on its enhanced end-systolic left ventricular pressure, stroke volume, cardiac output, ejection fraction, slope of ESPVR, and reduced end-systolic volume. Meanwhile, rolipram increased the systolic, diastolic blood pressure and pulse pressure without changing the arterial elastance which is proportional to the peripheral vascular resistance. Rolipram mismatched the rat normal ventricular-arterial coupling by reducing the normal value in control to the low level indicating that rolipram exerted positive inotropy with insignificant vascular effect in normal rats. However, the decreased effect on ventricular-arterial coupling by rolipram might be beneficial in heart failure of rats which have higher value of ventricular-arterial coupling (Cheng et al., 2008; Ky et al., 2013).

The *in vivo* positive inotropic effect of rolipram is under the condition of basal β-adrenergic tension by neurohumoral regulation. To elucidate whether this positive inotropic effect is independent of basal β-adrenergic stimulation, the isolated rat heart perfusion experiment was used. The results demonstrated that rolipram significantly enhanced rat left ventricular developed pressure, heart rate, and +dp/dt_max_ *in vitro* in a concentration-dependent manner. This effect occurred in both non-paced and paced models, and was independent of heart rate as reported (Beca et al., 2011). The increased inotropy by rolipram indicated that there is intrinsic cAMP content which exists in subcellular compartmentation independent of β-adrenergic stimulation.

To investigate the mechanism underlying the positive inotropic effect, the Ca^2+^ transient recording of rat left ventricular myocyte was used to analyze the effect of rolipram on Ca^2+^ handling. Ca^2+^ transient is composed of a fast rise phase mediated by Ca^2+^ release from SR to cytoplasm *via* RyR2 and a recovery phase or decaying phase by mainly re-uptaking Ca^2+^ from cytoplasm to SR *via* SERCA2a and extruding Ca^2+^ from cytoplasm to outside cell *via* NCX and PMCA. Rolipram increased the amplitude of Ca^2+^ transient induced by field stimulation without changing the diastolic Ca^2+^ level. The shape and amplitude of Ca^2+^ transient are determined mainly by RyR2, SERCA2a, NCX, and PMCA. First, the decaying phase was fitted by two exponential equations of type *y* = A*e^−αt^ + B*e^−βt^, which follows the method of concentration-time curve fitting following intravenous administration of a drug in human. The decaying phase fast component rate constant of “α” represents the activity of SERCA2a and slow one of “β” represents the activity of combined NCX and PMCA. Rolipram significantly increased the SERCA2a activity and decreased the combined NCX and PMCA activity based on their decaying rate constants’ changes. Second, to isolate the decreased activities of NCX and PMCA by rolipram in field stimulation-induced Ca^2+^ transient, caffeine-induced Ca^2+^ transient experiments which inactivate SERCA2a activity were used in conditions of normal Tyrode’s solution or Na^+^ and Ca^2+^-free extracellular solution. The results indicated that rolipram failed to change the rate constants of activities of combined NCX and PMCA or PMCA alone. This result apparently differed from the fitting data in field stimulation-induced Ca^2+^ transient experiment in which rolipram reduced the combined activities of NCX and PMCA. The discrepancy might be due to that the robust increased SERCA2a activity compressed the abilities of NCX and PMCA to extrude the Ca^2+^ to outside cell during the relaxation in field stimulation-induced Ca^2+^ transient and the direct effect of rolipram on activities of NCX and PMCA in caffeine-induced Ca^2+^ transient remained unchanged. This explanation also helps to understand the mechanism underlying the increased SR Ca^2+^ content by rolipram, in which SERCA2a re-uptaked more Ca^2+^ into to SR by rolipram and passively reduced Ca^2+^ extrusion to outside cell *via* NCX and PMCA by reducing cytoplasmic diastolic Ca^2+^ concentration without enhancing Ca^2+^ entry from outside cell *via* L-type Ca^2+^ channel (Kerfant et al., 2007; Beca et al., 2011). Third, rolipram enhanced the Ca^2+^ leak rate in the caffeine-induced Ca^2+^ transient experiment. However, diastolic Ca^2+^ level was not changed in field stimulation-induced Ca^2+^ transient experiment. The balancing effects by enhanced re-uptaking function of SERCA2a and increased Ca^2+^ leak might explain these data. The Ca^2+^ leak occurs during the relaxation period and is easily measured by caffeine-induced Ca^2+^ transient experiment in the condition of inactivating SERCA2a (caffeine widely opens RyR2 and make SR membrane has no effect on building Ca^2+^ concentration gradient) and NCX (0 Na^+^ and 0 Ca^2+^ were added to external solution to abolish NCX activity). However, RyR2 activity which mediates the Ca^2+^ transient rise phase was not analyzed due to the limitation in this study with slow sampling of 10 ms. Even though, the peak shape of field stimulation induced-Ca^2+^ transient demonstrated that there was a sharp peak in control and a blunt one in rolipram-treated groups (data was not shown). The peak shape is determined by decay “tail” RyR2 Ca^2+^ release, Ca^2+^ leak, and start of SERCA2a activity. The underlying mechanism still needs further investigation. Many studies have reported that PDE4 inhibition increased the PLB phosphorylation which enhances the activity of SERCA2a and RyR2 phosphorylation level (Kerfant et al., 2007). Moreover, the inhibition of PDE4 by rolipram increased the NCX current which was used to determine the Ca^2+^ content of cytoplasm (Kerfant et al., 2007; Beca et al., 2011). In our study, we measured the combined rate constant of NCX and PMCA activities and not NCX current. Furthermore, the direct effect of rolipram on NCX might be not equal to one in the global effect with the involvement of SERCA2a.

Although this study addressed the inotropic effects of rolipram both in *in vivo* and *in vitro* and its underlying mechanism was mediated by enhancing the SERCA2a activity, however, there are still a few limitations in this study. First, PDE4 constitutes a large fraction of the total PDE activity in rodent heart than in human heart (Eschenhagen, 2013), suggesting the data in this study should be applied to humans cautiously. Secondly, the mechanism of enhanced SERCA2a activity by rolipram still needs to be further explored, such as involvement with p-phospholamban mediated by PKA, CaMKII, protein phosphatase, etc.

In conclusion, rolipram, a PDE4 inhibitor, exerted positive inotropic effect both *in vivo* and *in vitro* without insignificant peripheral arterial elastance, which resulted in the mismatched ventricular-arterial coupling in normal rats. The inotropic effect was mediated by Ca^2+^ handling in which rolipram enhanced SERCA2a activity and Ca^2+^ leak and reduced NCX and PMCA activities. However, rolipram had no direct effects on rate constants of NCX and PMCA. These results indicated that this PDE4 inhibitor may serve as a potential positive inotropic agent for the treatment of heart failure.

## Data Availability

This manuscript contains previously unpublished data. The name of the repository and accession number are not available.

## Author Contributions

LC, HH, and MX contributed to study design. WL, RW, and KC carried out literature research. HH, MX, LG, WZ, XZ, and YW performed experiments. HH and MX contributed to data analysis and photographs analysis. LC contributed to manuscript preparation, and MB contributed to manuscript revision.

### Conflict of Interest Statement

The authors declare that the research was conducted in the absence of any commercial or financial relationships that could be construed as a potential conflict of interest.
